# Clinical outcomes after implantation of a sutureless aortic bioprosthesis with concomitant mitral valve surgery: the SURE-AVR registry

**DOI:** 10.1186/s13019-021-01523-w

**Published:** 2021-05-30

**Authors:** Max Baghai, Mattia Glauber, Raphael Fontaine, Jose Cuenca Castillo, Antony H. Walker, Ugolino Livi, José Montiel, Alistair Royse, Gianluigi Bisleri, Davide Pacini, Vincenzo Argano, Aurelien Roumy, George Asimakopoulos, Marco Solinas, on behalf of the SURE-AVR investigators, Marco Solinas, Raphael Fontaine, Max Baghai, Jose Cuenca Castillo, Antony H. Walker, Ugolino Livi, Josep Maria Padrò, Alistair Royse, Gianluigi Bisleri, Davide Pacini, Vincenzo Argano, Aurelien Roumy, George Asimakopoulos, Mattia Glauber

**Affiliations:** 1 Department of Cardiovascular Medicine, King’s College Hospital, Brixton, London, SE5 9RS UK; 2 U.O. Cardiochirurgia Mininvasiva, Istituto Clinico Sant’Ambrogio, Milan, Italy; 3 Chirurgie Cardiaque CHR Namur, Namur, Belgium; 4 Cirugía Cardiaca, Hospital Juan Canalejo, A Coruña, Spain; 5 Cardiothoracic Surgery, Blackpool Teaching Hospitals NHS Foundation Trust, Blackpool, UK; 6 Cardiochirurgia, Az. Osp. Univ. S. Maria della Misericordia, Udine, Italy; 7 Cirurgia Cardiaca, Hospital de la Santa Creu i Sant Pau, Barcelona, Spain; 8 Cardiovascular department, The University of Melbourne, Melbourne, Australia; 9 Division of Cardiac Surgery, University of Toronto, Toronto, Canada; 10 Cardiochiurgia, Policlinico Sant’Orsola, Bologna, Italy; 11 U.O.C. Cardiochirurgia, AOU Policlinico “Paolo Giaccone”, Palermo, Italy; 12 Service de chirurgie cardio-vasculaire, Centre Hospitalier Universitaire Vaudois, Lausanne, Switzerland; 13 Heart Surgery, Royal Brompton Hospital, London, UK; 14 U.O.C Cardiochirurgia Adulti, Ospedale del Cuore G. Pasquinucci, Fondazione Toscana Fabriele Monasterio, Massa, Italy

**Keywords:** Aortic valve stenosis, Aortic valve replacement, Aortic valve, Valvular disease

## Abstract

**Background:**

Early treatment of aortic valve stenosis is recommended in eligible symptomatic patients with severe aortic valve stenosis who would otherwise have a poor prognosis. The sutureless aortic valve bioprosthesis offers an alternative to standard aortic valve replacement with a sutured valve, but limited data are available in patients who have undergone multiple valve procedures involving the new, sutureless technology. We sought to investigate outcomes in high operative risk patients with previous or concomitant valve surgery who were implanted with a sutureless valve.

**Methods:**

SURE-AVR is an ongoing, prospective, multinational registry of patients undergoing aortic valve replacement. In-hospital and post-discharge outcomes up to 5 years were collected.

**Results:**

The study population comprised 78 patients (mean ± SD: age 73.6 ± 7.6 years, logistic EuroSCORE 18.0 ± 17.5) enrolled at 13 sites who presented for concomitant or previous mitral valve repair (*n* = 45) or replacement (*n* = 33), with or without additional concomitant procedures, and were implanted with a sutureless valve. Mean ± SD overall aortic cross-clamp time was 109 ± 41 min and cardiopulmonary bypass time was 152 ± 49 min. Mean ± SD aortic pressure gradients decreased from 37.6 ± 17.7 mmHg preoperatively to 13.0 ± 5.7 mmHg at hospital discharge, and peak aortic pressure gradient from 61.5 ± 28.7 to 23.4 ± 10.6 mmHg. Early events included 1 death, 1 transient ischaemic attack, and 1 bleed (all 1.3%); a permanent pacemaker implantation was required in 6 patients (7.7%), and 2 reoperations (not valve related) (2.6%) took place. Over a median follow-up of 55.5 months (Q1 13.4, Q3 68.6), 12 patients died (6 cardiovascular and 6 non-cardiovascular, both 2.1% per patient-year). Five-year survival was 81.3%. Late paravalvular leak occurred in 2 patients (0.7% per patient-year) and permanent pacemaker implantation was required in 3 patients (0.1% per patient-year). There was no apparent rise in mean or peak aortic pressure gradient over the study.

**Conclusions:**

These results suggest that the sutureless implant is a technically feasible procedure during mitral surgery and is associated with good clinical outcomes.

## Background

Aortic valve stenosis is the most frequent valvular disease resulting in surgical or catheter intervention in Western countries, and the prevalence is rising due to the ageing population [1]. Early treatment is recommended in eligible symptomatic patients with severe aortic valve stenosis, who would otherwise have a poor prognosis [1].

The sutureless aortic valve bioprosthesis offers an alternative to standard aortic valve replacement (AVR) with a sutured valve. It has a shorter cross-clamp time than traditional valves, and hence a shorter myocardial ischaemia time [2], and is associated with better clinical outcomes and reduced hospital costs [3]. In a meta-analysis involving 84 studies, reductions in aortic cross-clamp and cardiopulmonary bypass times were reported with the Perceval sutureless valve (LivaNova plc, London, UK) versus a traditional sutured prosthesis.

Limited data, restricted to small, single-centre, short-term cohorts, are available in patients who have undergone multiple valve procedures involving the new, sutureless technology [4–8]. In elderly high-risk patients with isolated AVR, rates of operative mortality range from 0 to 3% for sutureless AVR and from 4 to 10% for conventional AVR [2, 9–12]. However, higher rates have been reported in patients undergoing complex concomitant procedures (5.3–7.4% for sutureless AVR and 15.6% for conventional AVR) [7, 13]. We therefore sought to investigate early and late outcomes in high operative risk patients with previous or concomitant valve surgery who were implanted with the sutureless valve, using data from the Sorin Universal REgistry on Aortic Valve Replacement (SURE-AVR).

## Patients and methods

SURE-AVR is an ongoing, prospective, observational registry being conducted at 60 sites in 18 countries in Europe, Canada, and Australia (ClinicalTrials.gov identifier: NCT02679404). Patients implanted with any of the commercially available LivaNova aortic products are eligible for enrolment. This analysis focuses on patients implanted with the Perceval sutureless aortic valve who presented at 13 sites for concomitant or previous mitral valve repair or replacement with or without additional concomitant procedures.

The Perceval (LivaNova plc, London, UK) sutureless valve is a self-anchoring, self-expanding, surgical aortic bioprosthesis indicated for the replacement of damaged or malfunctioning native aortic heart valves or prostheses. It has a functional component, comprising bovine pericardium, stabilized in buffered glutaraldehyde solution, and a super-elastic metal alloy stent, which has the dual role of valve support and anchoring to the aortic root with no permanent sutures. Before implantation, the prosthesis diameter is reduced to a suitable size for loading onto a holder accessory. The valve is then positioned and released in the aortic root and post-dilated using a balloon catheter. The device is available in 4 sizes (small, medium, large, extra-large, corresponding to prosthesis heights of 31.0, 33.0, 35.5, and 37.5 mm, respectively). Implantation could be performed using a traditional surgical approach [14, 15] or through minimally invasive cardiac surgery [16–18].

Patients were enrolled in a sequential and prospective manner and were treated based on the standard of care at participating sites. All patients received a unique registry number in the electronic data capture system to guarantee their anonymity. Participation was voluntary, and patients could withdraw from participating at any time.

Baseline data were entered into an electronic case report form by trained study coordinators, and included demographic, clinical, echocardiographic, and surgical data. Follow-up visits were performed according to the centres’ usual practices (by telephone call, referring physician, or clinical visit) at 1 year and annually thereafter to 5 years, with follow-up at 7 and 10 years in selected centres. The results presented herein are limited to 5-year follow-up.

An electronic data capture system was used to allow specific quality control checks. The sponsor’s project team also applied checks to ensure an appropriate level of quality and compliance of the data. No source verification visits were conducted. Before enrolling patients, staff at each participating centre underwent training on the protocol, electronic case report form, and electronic data capture system.

The design and conduct of the registry are overseen by a study coordinating committee comprising representatives from the different regions involved in the study. The committee is responsible for registry oversight, including study progress, patient safety, and data quality and integrity.

### Clinical outcomes

Data on multiple procedural and hospital discharge variables were collected, including implant success, cross-clamp time, length of stay in the intensive care unit, and total length of hospitalization. Clinical success was defined as a successful valve implantation without the occurrence of major adverse events by the time of discharge. Major adverse events (investigator reported) were defined as death (all-cause, non-cardiovascular, and cardiovascular [including deaths of unknown cause]), stroke, and reintervention (surgery or other cardiac invasive therapy). Serious valve-related adverse events included bleeding, thromboembolism, valve thrombosis, endocarditis, non-structural dysfunction, and structural valve deterioration. All adverse events are defined according to the Valve Academic Research Consortium-2 criteria [19]. Severity of valve dysfunction was classified as mild (grade 1+), moderate (2+), moderate to severe (3+) or severe (4+). Improvement in clinical status was defined as an improvement of ≥1 in the New York Heart Association (NYHA) classification from baseline (before the procedure), measured annually throughout the study. Echocardiographic and haemodynamic data were collected. Early outcomes were defined as those occurring up to 30 days after the procedure and late outcomes as those occurring > 30 days after the procedure.

### Statistical analysis

Variables are described as mean ± standard deviation (SD) or median (quartile [Q1, Q3]; range) for quantitative variables and as number (%) for qualitative variables. Outcomes are reported as descriptive statistics. The rates of early adverse events were calculated as the number of events divided by the number of patients. Linearized complication rates were calculated as the number of late events divided by the number of patient-years. The statistical analyses were performed using SAS® (Release 9.4, by SAS Institute Inc., Cary, NC, USA).

## Results

A total of 1134 patients (482 men and 652 women) implanted with a sutureless valve were prospectively enrolled in the SURE-AVR registry between March 2011 and March 2019. Of these, 78 patients (enrolled at 13 sites) had undergone a previous and/or concomitant mitral valve procedure: 45 mitral valve repair and 33 mitral valve replacement.

The characteristics of the study population are detailed in Table 1. Most patients were female (74.4%) and the mean ± SD age at surgery was 73.6 ± 7.6 years. The main indication for a sutureless implant was aortic valve stenosis (59.0%). A diagnosis of mitral regurgitation was reported for 64.1% of the patients. Tricuspid regurgitation was diagnosed in 61.5% of the patients. Most of the patients (87.0%) were in NYHA class II or III. Mean ± SD logistic EuroSCORE I was 18.0 ± 17.5.

**Table 1:** Baseline demographics and clinical characteristics in overall population and according to type of mitral valve surgery

Characteristic	Overall population (***n*** = 78)	Mitral repair (***n*** = 45)	Mitral replacement (***n*** = 33)
Women, n (%)	58 (74.4)	31 (68.9)	27 (81.8)
Age at surgery (years), mean ± SD	73.6 ± 7.6	73.0 ± 8.3	74.5 ± 6.5
Range	55–88	55–88	60–87
Body surface area (m^2^), mean ± SD	1.7 ± 0.2	1.8 ± 0.2	1.7 ± 0.2
Diagnosis, n (%)			
Aortic valve stenosis	46 (59.0)	28 (62.2)	18 (54.5)
Aortic steno-regurgitation	16 (20.5)	7 (15.6)	9 (27.3)
Aortic regurgitation	15 (19.2)	10 (22.2)	5 (15.2)
Mitral stenosis	7 (9.0)	3 (6.7)	4 (12.1)
Mitral steno-regurgitation	10 (12.8)	2 (4.4)	8 (24.2)
Mitral regurgitation	50 (64.1)	36 (80.0)	14 (42.4)
Tricuspid regurgitation (mild to severe)	48 (61.5)	28 (62.2)	20 (60.6)
Risk factors, n (%)			
Diabetes mellitus	23 (29.5)	15 (33.3)	8 (24.2)
Coronary artery disease	28 (35.9)	16 (35.6)	12 (36.4)
Endocarditis	6 (7.7)	3 (6.7)	3 (9.1)
Renal insufficiency	12 (15.4)	7 (15.6)	5 (15.2)
Cerebrovascular events	4 (5.1)	4 (8.9)	0
Chronic lung disease	9 (11.5)	7 (15.6)	2 (6.1)
NYHA class	(*n* = 77)	(*n* = 45)	(*n* = 32)
I	1 (1.3)	1 (2.2)	0
II	35 (45.5)	24 (53.3)	11 (34.4)
III	32 (41.6)	15 (33.3)	17 (53.1)
IV	9 (11.7)	5 (11.1)	4 (12.5)
Left ventricular ejection fraction (%)	(*n* = 25)	(*n* = 15)	(*n* = 10)
Mean ± SD	53.1 ± 9.6	50.4 ± 8.8	57.0 ± 9.7
Median (quartile 1, quartile 3)	55.0 (45.4; 60.0)	53.7 (45.0; 57.0)	57.5 (50.0; 62.0)
Logistic EuroSCORE I	(*n* = 50)	(*n* = 29)	(*n* = 21)
Mean ± SD	18.0 ± 17.5	15.9 ± 17.7	20.9 ± 17.3
Median (quartile 1, quartile 3)	11.5 (7.5; 21.5)	9.1 (6.6; 18.5)	15.7 (9.1; 24.4)

*NYHA* New York Heart Association, *SD* standard deviation

Before the sutureless implant, 11.5% of patients had undergone aortic valve replacement, 11.5% mitral valve replacement, 10.3% coronary artery bypass graft (CABG), and 7.7% mitral valve repair (Table 2).

**Table 2:** Previous cardiac surgery in the overall population and according to type of mitral valve surgery^a^

Surgery, n (%)	Overall population (***n*** = 78)	Mitral repair (***n*** = 45)	Mitral replacement (***n*** = 33)
Coronary artery bypass graft	8 (10.3)	1 (2.2)	7 (21.2)
Repair procedure	10 (12.8)	5 (11.1)	5 (15.2)
Aortic valve repair	5 (6.4)	3 (6.7)	2 (6.1)
Mitral valve repair	6 (7.7)	3 (6.7)	3 (9.1)
With ring	3/6 (50.0)	2/3 (66.7)	1/3 (33.3)
Valve replacement	15 (19.2)	3 (6.7)	12 (36.4)
Aortic valve replacement	9 (11.5)	3 (6.7)	6 (18.2)
Mitral valve replacement	9 (11.5)	0	9 (27.3)

^a^Some patients may have undergone more than one previous cardiac surgery

### Surgical procedures

The majority (76.9%) of patients underwent a median sternotomy approach and a large-size valve was implanted in 46.2% (Table 3). A concomitant procedure was reported in 89.7% of the patients, with CABG performed in 16.7% and tricuspid repair in 16.7%. The sutureless valve was successfully implanted in all patients.

**Table 3:** Procedural characteristics in the overall population and according to type of mitral valve surgery

Characteristic, n (%)	Overall population (***n*** = 78)	Mitral repair (***n*** = 45)	Mitral replacement (***n*** = 33)
Surgical approach			
Median sternotomy	60 (76.9)	30 (66.7)	30 (90.9)
Minimally invasive cardiac surgery	18 (23.1)	15 (33.3)	3 (9.1)
Mini thoracotomy	17 (21.8)	15 (33.3)	2 (6.1)
Mini sternotomy	1 (1.3)	0	1 (3.0)
Valve size (aortic annulus diameter)			
Small (19–21 mm)	9 (11.5)	5 (11.1)	4 (12.1)
Medium (21–23 mm)	29 (37.2)	14 (31.1)	15 (45.5)
Large (23–25 mm)	36 (46.2)	23 (51.1)	13 (39.4)
Extra large (25–27 mm)	4 (5.1)	3 (6.7)	1 (3.0)
Concomitant procedures	70 (89.7)	42 (93.3)	28 (84.8)
CABG	13 (16.7)	11 (24.4)	2 (6.1)
Atrial fibrillation ablation	3 (3.8)	2 (4.4)	1 (3.0)
Mitral surgery	69 (88.5)	42 (93.3)	27 (81.8)
Repair procedure	43/69 (62.3)	42/42 (100.0)	1/27 (3.7)
Replacement procedure	26/69 (37.7)	0	26/27 (96.3)
Tricuspid surgery	13 (16.7)	6 (13.3)	7 (21.2)
Repair procedure	13/13 (100.0)	6/6 (100.0)	7/7 (100.0)
Replacement procedure	0	0	0
Implant successful	78 (100.0)	45 (100.0)	33 (100.0)
Cross-clamp time (min)			
Mean ± SD	109 ± 41	107 ± 28	113 ± 55
Median (quartile 1, quartile 3)	105 (84; 132)	105 (85; 125)	106 (77; 136)
Cardiopulmonary bypass time (min)			
Mean ± SD	152 ± 49	147 ± 32	159 ± 68
Median (quartile 1, quartile 3)	145 (121; 173)	141 (124; 168)	148 (118; 191)

*CABG* coronary artery bypass graft, *SD* standard deviation

Mean ± SD overall aortic cross-clamp time was 109 ± 41 min (median 105, Q1 84, Q3 132 min) and the mean ± SD cardiopulmonary bypass time was 152 ± 49 min (median 145, Q1 124, Q3 173 min) (Table 3). Mean length of stay in the intensive care unit was 3.1 ± 4.1 days (median 2.0, Q1 1.0, Q3 3.5 days); total length of hospital stay was 14.2 ± 9.4 days (median 11.0, Q1 8.0, Q3 16.0 days).

### Early outcomes

**Fig. 1:**
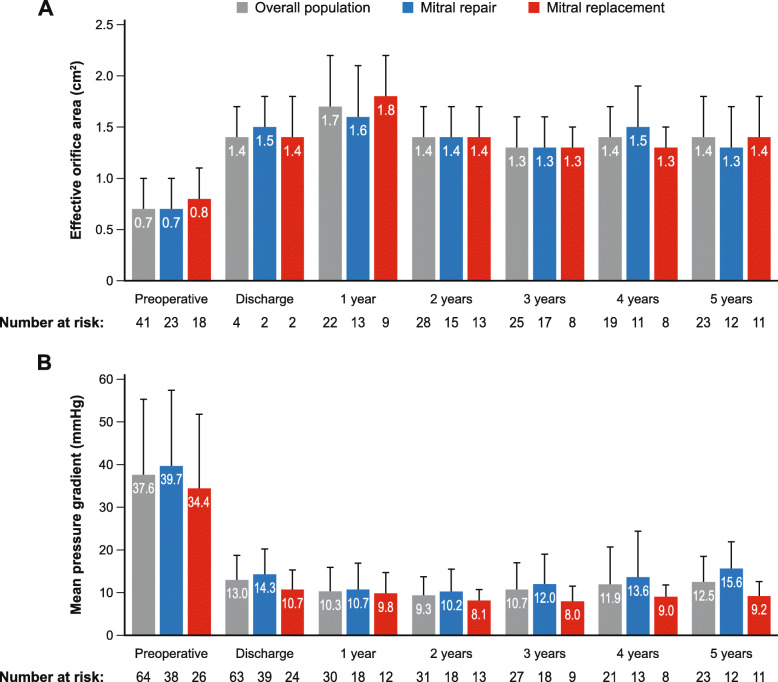
**a** Effective orifice area and **b** mean aortic pressure gradient, preoperatively and up to 5 years of follow-up. Vertical lines indicate standard error

Mean ± SD effective orifice area was 0.7 ± 0.3 cm^2^ before surgery and 1.4 ± 0.3 cm^2^ at discharge (Fig. 1). Mean aortic pressure gradient decreased from 37.6 ± 17.7 mmHg preoperatively to 13.0 ± 5.7 mmHg at discharge from hospital, and peak aortic pressure gradient from 61.5 ± 28.7 mmHg to 23.4 ± 10.6 mmHg.

Early outcomes are detailed in Table 4. There was 1 death (due to septicaemia), 1 transient ischaemic attack, and 1 case of bleeding (all 1.3%). There were no cases of endocarditis, myocardial infarction, or structural valve deterioration. Mild non-structural valve dysfunction (not requiring intervention) was reported in 10 patients (12.8%). Mild paravalvular leak occurred in 1 patient (1.3%). A permanent pacemaker implantation was required in 6 patients (7.7%), due to third-degree atrioventricular block in 4 patients and for unspecified conduction disorders in 2 patients. Two reoperations (not valve related) (2.6%) took place, 1 due to subtamponade pericardial effusion and 1 to superior vena cava syndrome. No explants were reported.

**Table 4:** Early and late adverse events in the overall population and according to type of mitral valve surgery

Event	Early events (≤30 days), n (%)			Late events (> 30 days), n (rate per 100 patient-years)		
	Overall population (***n*** = 78)	Mitral repair (***n*** = 45)	Mitral replacement (***n*** = 33)	Overall population (***n*** = 78; 282.5 patient-years)	Mitral repair (***n*** = 45; 160.6 patient-years)	Mitral replacement (***n*** = 33; 122.2 patient-years)
Death	1 (1.3)	0	1 (3.0)	12 (4.2)	6 (3.7)	6 (4.9)
Cardiovascular	0	0	0	6 (2.1)	3 (1.9)	3 (2.5)
Non-cardiovascular	1 (1.3)^a^	0	1 (3.0)	6 (2.1)^b^	3 (1.9)	3 (2.5)
Reintervention	2 (2.6)	0	2 (6.1)	4 (1.4)	3 (1.9)	1 (0.8)
Valve related	0	0	0	3 (1.1)^c^	2 (1.2)	1 (0.8)
Not valve related	2 (2.6)	0	2 (6.1)	1 (0.4)	1 (0.6)	0
Cerebrovascular accident	1 (1.3)	1 (2.2)	0	2 (0.7)	1 (0.6)	1 (0.8)
Stroke	0	0	0	1 (0.4)^d^	0	1 (0.8)
Transient ischaemic attack	1 (1.3)	1 (2.2)	0	1 (0.4)	1 (0.6)	0
Bleeding	1 (1.3)	0	1 (3.0)	2 (0.7)	2 (1.2)	0
Minor^e^	0	0	0	1 (0.4)	1 (0.6)	0
Major^f^	0	0	0	0	0	0
Not classified	1 (1.3)	0	1 (3.0)	1 (0.4)	1 (0.6)	0
Non-structural valve dysfunction	10 (12.8)	1 (2.2)	9 (27.3)	13 (4.6)	8 (5.0)	5 (4.1)
Intra-prosthetic regurgitation (1+)	6 (7.7)	0	6 (18.2)	9 (3.2)	6 (3.7)	3 (2.5)
Paravalvular leak	1 (1.3)	1 (2.2)	0	2 (0.7)	1 (0.6)	1 (0.8)
Paravalvular leak plus intra-prosthetic leak (no haemodynamic consequences)	3 (3.8)	0	3 (9.1)	2 (0.7)	1 (0.6)	1 (0.8)
Permanent pacemaker implant, due to:	6 (7.7)	3 (6.7)	3 (9.1)	3 (1.1)	1 (0.6)	2 (1.6)
Atrioventricular block (third degree)	4 (5.1)	3 (6.7)	1 (3.0)	1 (0.4)	0	1 (0.8)
Other reason	2 (2.6)	0	2 (6.1)	2 (0.7)	1 (0.6)	1 (0.8)

^a^ Due to septicaemia

^b^ Due to neoplasia (3 patients) in mitral replacement group, renal failure (1 patient) and non − valve-related death (2 patients) in mitral repair group

^c^ 1 endocarditis, 2 non-structural valve disease

^d^ Non-disabling stroke

^e^ Any bleeding worthy of clinical mention (e.g. access site haematoma) that does not qualify as life-threatening, disabling, or major [19]

^f^ Overt bleeding either associated with a drop in the haemoglobin level of ≥3.0 g/dL or requiring transfusion of 2 or 3 units of whole blood/RBC, or causing hospitalization or permanent injury, or requiring surgery AND does not meet criteria of life-threatening or disabling bleeding [19]

*AE* adverse event

### Late outcomes

Median study follow-up duration was 55.5 months (Q1 13.4, Q3 68.6 months), with a cumulative follow-up of 289.2 patient-years (including 282.5 patient-years). Late outcomes are detailed in Table 4. Mean effective orifice area was 1.4 ± 0.4 cm^2^, mean aortic pressure gradient was 12.5 ± 6.0 mmHg, and peak aortic pressure gradient was 21.4 ± 10.7 mmHg at 5 years (Fig. 1).

**Fig. 2:**
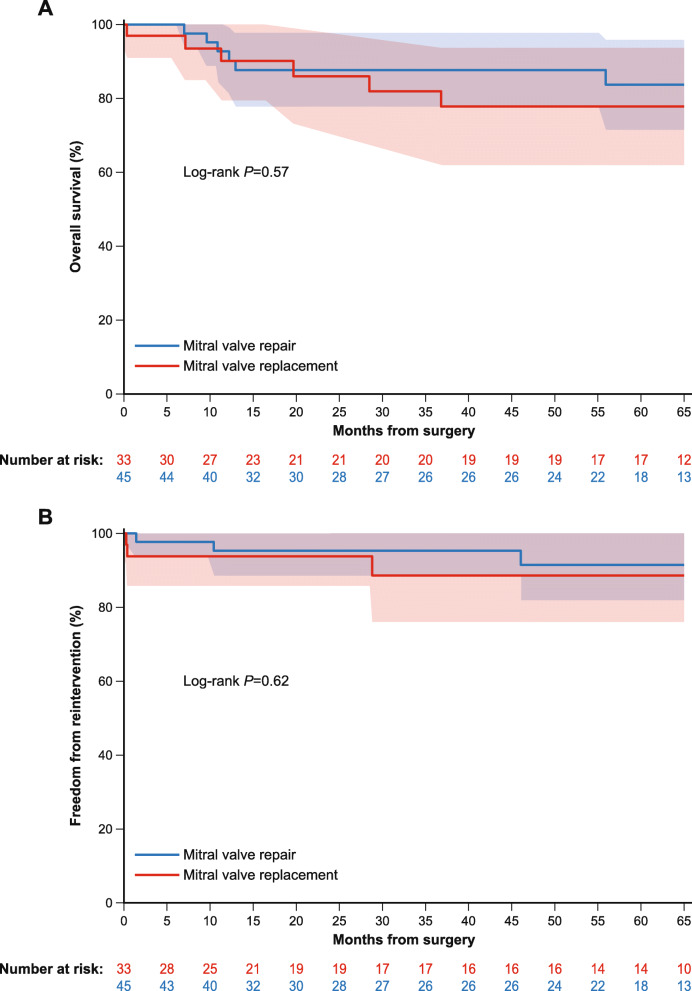
**a** Overall survival and **b** freedom from reintervention in patients who underwent mitral repair or mitral replacement

Six cardiovascular deaths (2.1% per patient-year) were reported as late outcomes, 1 caused by worsening heart failure, 1 due to a possible cardiac cause, and 4 of an unknown cause. Six non-cardiovascular deaths (2.1% per patient-year) occurred, 3 as a result of neoplasia, 2 due to other non − valve-related causes (1 urosepsis, 1 unknown [the patient died while abroad and based on the information provided, their doctor classified the death as non-cardiovascular]), and 1 due to renal failure. Survival at 5-year follow-up was 81.3% in the overall population, 83.8% in the mitral repair group, and 77.9% in the mitral replacement group (Fig. 2). Two cases of bleeding (0.7% per patient-year) and 1 case each of transient ischaemic attack, non-disabling stroke, and endocarditis (all 0.4% per patient-year) occurred. No cases of myocardial infarction, thrombosis, or structural valve deterioration were reported. In addition to the 2 cases requiring re-intervention described above, mild non-structural valve dysfunction (intra-prosthetic regurgitation) was reported in 9 patients (3.2% per patient-year), none of which required intervention. Paravalvular leak occurred in 2 patients (0.7% per patient-year) (mild regurgitation without any haemodynamic consequences). Permanent pacemaker implantation was required in 3 patients (1.1% per patient-year), due to atrioventricular block III in 1 patient and other unspecified conduction disorders in 2 patients. A transcatheter valve (0.4% per patient-year) was implanted in 1 patient after 873 days, due to moderate/severe regurgitation. Two sutureless valves were explanted (0.7% per patient-year), 1 due to endocarditis and 1 to non-structural valve dysfunction. There was no apparent rise in either the mean aortic or peak gradient over the course of the study (Fig. 1).

## Discussion

In this population of patients at high operative risk who had undergone a previous and/or concomitant valvular procedure, the sutureless biological aortic valve was a technically feasible procedure during mitral surgery and was associated with good clinical outcomes, both early after the procedure and up to 5 years of follow-up. Implantation of the sutureless aortic valve in the presence of a previously implanted prosthesis in the mitral position or with a concomitant mitral valve procedure appears to be a viable option.

Limited comparative data are currently available in patients undergoing multiple valve procedures with stented or sutureless valves. One of the largest is the Epic study [20], which enrolled an elderly all-comer population undergoing aortic, mitral, or double-valve replacement with a stented porcine xenograft during 2001 to 2012 and followed them for a mean ± SD of 4.5 ± 3.5 years. Of 2544 patients enrolled, 347 underwent mitral valve replacement, 27.6% of whom had undergone previous cardiac surgery and 39.6% were undergoing additional cardiac procedures. More recent studies are available, comparing sutureless with conventional AVR in patients undergoing concomitant cardiac procedures, or reporting outcomes in patients undergoing isolated sutureless AVR or with concomitant cardiac procedures, but most are relatively small, retrospective in design, have no comparator arm, or involve lower-risk patients [7, 13, 21]. In the recently published prospective, randomized PERSIST-AVR study [22], 910 patients with severe symptomatic aortic valve stenosis were randomized to undergo implantation with a sutureless valve or a standard aortic valve, using a conventional or minimally invasive approach. The study showed that in patients undergoing isolated AVR or AVR plus CABG, the sutureless valve reduced operative time and was noninferior to conventional bioprostheses for 1-year major complications (a composite of all-cause death, myocardial infarction, stroke, or valve reintervention). The rate of concomitant surgical procedures in the PERSIST-AVR study was, however, much lower than in our present study (30% vs 89.7%, respectively),

Prolonged aortic cross-clamping during AVR is associated with higher mortality and morbidity in patients undergoing cardiac surgery [23]. Flameng et al. [2], in a population of 32 patients (median logistic EuroSCORE 9.99) requiring AVR with or without concomitant CABG, reported that a sutureless stent-mounted aortic valve could be implanted in less than 20 min of aortic cross-clamping, and in 23 min for AVR plus CABG. In a retrospective study of 314 patients undergoing AVR with a sutureless bioprosthesis (29.9% of whom underwent CABG), the mean ± SD aortic cross-clamp time was 39 ± 15 min for isolated AVR and 52 ± 26 min for AVR with concomitant CABG [13]. Hanedan et al. [7] reported longer operative times in their study of 70 high-risk elderly patients undergoing sutureless AVR plus other cardiac procedures (76.3% of whom underwent CABG), with a mean ± SD cross-clamp time of 78 ± 28 min and a cardiopulmonary bypass time of 119 ± 42 min, both of which were statistically significantly shorter than with conventional AVR using a sutured valve (122 ± 38 min and 166 ± 50 min, respectively; both *P* = 0.001). In the Epic study [20], the mean ± SD aortic cross-clamp time was 84 ± 44 min and the cardiopulmonary bypass time was 142 ± 60 min. The operative times were longer in the present study (cross-clamp 109 ± 41 min and cardiopulmonary bypass 152 ± 49 min), reflecting the high rate of mitral (88.5%) and tricuspid (16.7%) repair and replacement procedures in this complex and high-risk population, but still appeared shorter than reported for conventional aortic valve replacement [7]. Owing to the lack of a matched cohort, it is difficult to demonstrate a shorter operative time with the sutureless versus sutured valve.

In the present study, one patient (1.3%) died within 30 days of the procedure, which compares with 1.9% in a single-centre experience of 617 patients using the same sutureless valve [24]. Higher rates of 30-day death were reported by Hanedan et al. [7] (5.3% for sutureless AVR and 15.6% for conventional AVR). Rubino et al. reported a 1.4% in-hospital mortality rate after an isolated aortic valve procedure (1.4%), which increased to 7.4% when performed alongside CABG [13]. Five-year survival rates were markedly higher in our study than in Epic [20] (81.3% vs 39.3% with double valve replacement) whereas bleeding was similar (2.6% vs 2.9% after double valve replacement). Only 1 patient (1.3%) had a cerebrovascular accident (no strokes were reported) during the early postoperative period, compared with 9.1% with double valve replacement in Epic [20].

The need for permanent pacemaker implantation remains a concern with the use of sutureless valves, with postoperative incidence rates of 5% [24] and 10.8% [25]. The rate of permanent pacemaker implantation after transcatheter AVR with new-generation devices is highly variable (ranging from 2.3 to 36.1% in a recent systematic review [26]), and is influenced by electrical, anatomical, and procedural factors. In the current study, 6 patients (7.7%) underwent permanent pacemaker implantation within 30 days of the procedure, 4 due to third-degree atrioventricular block. By late follow-up, 3 further patients (1.1% per patient-year) had undergone pacemaker implantation (third-degree atrioventricular block: 0.4% per patient-year). Our results show a lower rate of pacemaker implantation compared with the Epic study, which reported an 11.6% rate during hospitalization after double valve replacement [20]. Our findings are consistent with a large study of 658 patients, in which the rate of pacemaker implantation at 1 year for third-degree atrioventricular block was 9.6% for the sutureless valve [21].

Haemodynamic data – in terms of effective orifice area and mean and peak aortic pressure gradients – were good and remained stable through 5 years of follow-up. Further, the rates of paravalvular leakage were low, comprising mild regurgitation with no haemodynamic consequences, and there was no apparent structural valve deterioration. These findings − consistent with a previous study in patients undergoing isolated sutureless aortic valve procedures [27] − are reassuring, as the current labelling for the Perceval sutureless valve includes a precaution about its use in concomitant mitral procedures.

### Limitations

This study is limited by the small population size, the heterogeneous population, the lack of a control group, and high enrolment at 1 centre.

## Conclusion

This study, in high operative risk patients implanted with a sutureless valve in the presence of a previously implanted prosthesis in the mitral position or with a concomitant mitral valve procedure, confirms that the sutureless implant is a technically feasible procedure during multivalvular surgery and is associated with good clinical outcomes.

## Data Availability

The datasets used and/or analysed during the current study are available from the corresponding author on reasonable request.

## Abbreviations

AVR: Aortic valve replacement
CABG: Coronary artery bypass graft
CI: Confidence interval
NYHA: New York Heart Association
Q: Quartile
SD: Standard deviation
SURE-AVR: Sorin Universal REgistry on Aortic Valve Replacement
